# Infants' Food

**Published:** 1890-03-01

**Authors:** 


### DIETETICS.

### INFANTS' DIET.

No food has yet been found so suitable for the young of all animals as their mother's milk. Some time ago, however, a Dr. Brouzet said, the State ought to interfere and prevent mothers from suckling their children, lest they should communicate disease! We are not, however, aware that it has ever been satisfactorily demonstrated that disease has been communicated by the medium of milk from the breast. Disease has been contracted from sores on the breast, but not from the milk. It is well known that the poor, thin milk of weakly mothers is not sufficiently nutritive to the child; but this is not the communication of disease by milk, into which fluid the obiquitous microbe does not appear to have yet penetrated. Unfortunately, all mothers are not able to nurse their children, and many other mothers, if able to do so, will not fulfil this natural function. Hence, probably, the demand for such preparations as Baron Leibig's food for infants, since supplemented by rivals, as Mellin's, Benger's, Savory and Moore's, with others too numerous to mention. But whatever food is given to infants, the premature weaning from good mothers' milk often results in consequences, patent enough to the medical eye although insiduous. While the external aspect of the child may be that of health, the muscles are not strong and the bones do not harden sufficiently early, if at all. When the child tries to walk, the limbs may give way, and rachitis or rickets occurs. From experiments on animals it would appear that rickets is certainly capable of being originated by improper feeding. And especially so in children, when, as is often the case, bread stuffs and meat are given at a time when the digestive organs are only adapted to assimilate milk. It is only when the teeth are on their way to the front, that the parotid gland secretes a fluid capable of commencing the digestion of bread stuffs. And it is only when the teeth have appeared that meat and other articles of diet requiring mastication should be given. As a rule, until the first teeth present, no other food than milk should be allowed. After this period some kind of malted food may be given. But it is not well to trust too much to malted food, for if we rear children habitually, without putting them to the necessity of digesting their food, we may evolve beings of weak digestion. And the vaunted attribute of malted food is stated to be, that starch has been so treated by chemical process, that the work of digestion has been partially performed. Malted foods should only be used as an introduction to farinaceous foods. And animal food should not be given until at least two back teeth have appeared. We have been led into the foregoing reflections by reading some remarks recently made by an American author (Dr. Louis Starr), who says (*Annual of the Universal Medical Sciences*, 1889): "There can be no doubt, although the statement is a bold one, and seemingly contrary to nature, that, taking the average, infants properly brought up by hand are better developed and enjoy more perfect health than those completely breast-fed. . . . For, unfortunately, the woman who has sufficient health and strength to furnish an abundant supply of good milk is unique in our day, and the great bulk of those who do nurse children grow pale, thin, and feeble, and give milk which, though sufficient in quantity to fill the suckling's stomach and satisfy the cravings of hunger, does not contain enough pabulum to satisfy the demand nutrition. Such mothers complain that their children are puny, peevish, and always ailing, and wonder why their neighbours' babies, fed upon the bottle, are so round, jolly, and healthy." This, of course, applies to American mothers, and we do not think it is so generally applicable to English mothers. Both climate and manner of life has much to do with the capability of women nursing their children. It is well known that in our tropical possessions—India, for instance—few women are able to nurse more than once or twice, in consequence of the debility produced by the heat, even if no illness occurs. Hence the fact of so many Anglo-Indian children being reared either by hand or on the milk of a native female. As the latter alternative is an expensive one, hand feeding, especially amongst European soldiers' children, is usually adopted. The mortality of Anglo-Indian children is enormous, and much of the death-rate may be attributed to hand-feeding. Now the climate of the Southerly United States is more or less tropical, and we are not surprised to learn from Dr. Louis Starr that women as a rule are unable to nurse their children. But we should scarcely have thought the same observation would have applied to the Northern States. Dr. Starr, however, evidently generalises. It has been advanced that if the population of the United States of America were not recruited by European blood the race would much degenerate, and possibly die out. The typical Yankee is certainly not the equal in physique to the typical Briton. And the average American girl although "vivacious, beautiful and rich," is not the equal in physique to the average British girl. Nor do we think that, as a rule, the American matron can compete with the British matron as regards the capability of nursing children. All this seems to the reflective mind somewhat favourable to the stability of Britain as a nation.

